# Impact of *Staphylococcus aureus* Small Colony Variants on Human Lung Epithelial Cells with Subsequent Influenza Virus Infection

**DOI:** 10.3390/microorganisms8121998

**Published:** 2020-12-15

**Authors:** Janine J. Wilden, Eike R. Hrincius, Silke Niemann, Yvonne Boergeling, Bettina Löffler, Stephan Ludwig, Christina Ehrhardt

**Affiliations:** 1 Institute of Virology Muenster (IVM), Westfaelische Wilhelms-University Muenster, 48149 Muenster, Germany; wildenja@ukmuenster.de (J.J.W.); hrincius@uni-muenster.de (E.R.H.); borgelin@uni-muenster.de (Y.B.); ludwigs@uni-muenster.de (S.L.); 2 Institute of Medical Microbiology, Westfaelische Wilhelms-University Muenster, 48149 Muenster, Germany; Silke.Niemann@uni-muenster.de; 3 Institute of Medical Microbiology, Jena University Hospital, 07747 Jena, Germany; bettina.loeffler@med.uni-jena.de; 4 Cluster of Excellence EXC 2051 “Balance of the Microverse”, FSU Jena, 07743 Jena, Germany; 5 Cluster of Excellence EXC 1003 “Cells in Motion”, WWU Muenster, 48149 Muenster, Germany; 6 Section of Experimental Virology, Institute of Medical Microbiology, Jena University Hospital, 07745 Jena, Germany

**Keywords:** *Staphylococcus aureus*, small colony variants, influenza virus, super-infection, pro-inflammatory response

## Abstract

Human beings are exposed to microorganisms every day. Among those, diverse commensals and potential pathogens including *Staphylococcus aureus* (*S. aureus*) compose a significant part of the respiratory tract microbiota. Remarkably, bacterial colonization is supposed to affect the outcome of viral respiratory tract infections, including those caused by influenza viruses (IV). Since 30% of the world’s population is already colonized with *S. aureus* that can develop metabolically inactive dormant phenotypes and seasonal IV circulate every year, super-infections are likely to occur. Although IV and *S. aureus* super-infections are widely described in the literature, the interactions of these pathogens with each other and the host cell are only scarcely understood. Especially, the effect of quasi-dormant bacterial subpopulations on IV infections is barely investigated. In the present study, we aimed to investigate the impact of *S. aureus* small colony variants on the cell intrinsic immune response during a subsequent IV infection in vitro. In fact, we observed a significant impact on the regulation of pro-inflammatory factors, contributing to a synergistic effect on cell intrinsic innate immune response and induction of harmful cell death. Interestingly, the cytopathic effect, which was observed in presence of both pathogens, was not due to an increased pathogen load.

## 1. Introduction

The respiratory tract is a major portal for microorganisms, through which virus infections can cause non-symptomatic, mild, and self-limiting but also severe diseases, sometimes with fatal outcomes [1]. A growing body of evidence shows that the human respiratory tract contains a highly adapted microbiota including commensal and opportunistic pathogens. Among those, *Staphylococcus aureus* (*S. aureus*) is of special importance, forming quasi-dormant subpopulations characterized by increased fitness compared to other phenotypes [2]. Colonization of *S. aureus* could either be persistent or non-persistent, whereby nasal colonization appears to be the most prominent localization [3]. *S. aureus* as a community-acquired pathogen is already colonized on approximately 30% of the human population, some without causing any symptoms [4]. During long-term colonization or infection, *S. aureus* can change phenotypes to so-called small colony variants (SCVs), which adapt in their metabolic and phenotypic characteristics, allowing them to evade the host’s immune system. SCVs can be localized intracellularly and are characterized by a slow growth rate, non-pigmentation, less hemolytic activity, and decreased antibiotic susceptibility [5,6,7] but often enhanced surface presentation of adhesion molecules [8]. SCVs are often misdiagnosed [9]. Due to their slow growth, they often get overgrown by other bacteria, and an initially effective antibiotic treatment results in the development of resistances accompanied by chronic and relapsing infections [5,6,8,10,11]. The clinical relevance of colonizing SCVs gets obvious in patients with chronic respiratory diseases, such as chronic obstructive pulmonary disease (COPD) or cystic fibrosis (CF) [5]. Patients who are colonized with bacteria are more likely to suffer from recurring infections [12], as the phenotype can revert to the pathogenic phenotype.

Besides, simultaneous occurrence of different pathogens can induce or even exacerbate a pathological effect in the lung. Super-infections with influenza viruses (IV) and with the community-acquired *S. aureus* are known to be harmful and lead to increased inflammatory lung damage [13]. Due to their quick adaptation and genomic changes, both pathogens can evade the host’s immune response, causing the tedious development of effective medications. Concerning super-infections, most studies describe infections with a primary viral infection that paves the path for a secondary bacterial infection [14,15,16,17]. However, there is evidence that primary bacterial colonization also occurs prior to viral infections [18].

However, the influence of colonizing *S. aureus* SCVs on subsequent IV infection is largely unexplored. Thus, in the present study, we aimed to investigate the effect of the bacterial strain *S. aureus* 3878_SCV_ on cell intrinsic immune responses to a subsequent IV infection, in vitro. Here, we observed that the response of anti-viral gene expression was barely changed. However, pro-inflammatory genes were highly upregulated upon super-infection, resulting in an induction of necrotic cell death. Thus, we were able to show that colonizing SCVs could enhance severity of subsequent viral infection.

## 2. Materials and Methods

### 2.1. Cell Lines, Virus Strains, and Bacteria Strain

All cell lines were cultivated at 37 °C and 5% CO_2_ under sterile conditions. Human lung epithelial cells A549 (American Type Culture Collection (ATCC), Wesel, Germany) were cultivated in Dulbeccos’s modified eagle medium (DMEM; Sigma-Aldrich, St. Louis, MO, USA) and Madin-Darby canine kidney cells II (MDCKII) in minimum essential medium eagle (MEM; Sigma-Aldrich, St. Louis, MO, USA), supplemented with 10% fetal bovine serum (FBS; Biochrom, Berlin, Germany).

The human IV strains A/Puerto Rico/8/34 (H1N1, PR8-M) and A/Panama/2007/99 (H3N2, Panama) were taken from the virus stock of the Institute of Virology Muenster, 48149 Muenster, Germany, subcultured and passaged on MDCKII cells.

The persisting bacterial strain *S. aureus* 3878_SCV_, wildtype phenotype strain *S. aureus* 3878_WT_, and the human lung isolate of another SCV phenotype strain *S. aureus* 814_SCV_ (provided by Karsten Becker, Institute of Medical Microbiology, Muenster, Germany) were stored at −80 °C in a 30% glycerol/brain-heart infusion (BHI; Merck; Darmstadt, Germany) medium. *S. aureus* 3878_SCV_ and *S. aureus* 3878_WT_ were already characterized and described previously [10,19,20,21]. Before experiments, bacteria were plated on blood agar plates to take single clones, which were inoculated in BHI medium and incubated for 24 h at 37 °C and 5% CO_2_. For bacterial infection, bacterial suspension was washed with phosphate buffered saline (PBS) (4000 rpm; 4 °C; 5 min) and adjusted to an optical density of OD_600nm_ = 1. Growth kinetics were performed to determine a colony forming unit (CFU) of 2 × 10^8^ CFU/mL at OD_600nm_ = 1 for each bacterial strain used.

### 2.2. Super-Infection Protocol

Human lung epithelial cells were seeded in either 6-well plates (0.5 × 10^6^) or 12-well plates (0.2 × 10^6^) in 2 mL or 1 mL culture medium 24 h before infection. For bacterial infection, the overnight culture was set to OD_600nm_ = 1 to determine the multiplicity of infection (MOI). Cells were washed with PBS and infected with *S. aureus* 3878_SCV_ in invasion media (DMEM_INV_: DMEM supplemented with 1% human serum albumin, 25 nmol/L HEPES) for 24 h with a MOI of 0.01. For viral infection, supernatant was aspirated, cells were washed with PBS and incubated with IV PR8-M (MOI = 0.1) or IV Panama (MOI = 0.01) in infection PBS (PBS_INF_: PBS supplemented with 0.2% bovine serum albumin (BSA), 1 mM MgCl_2_, 0.9 mM CaCl_2_, 100 U/mL penicillin, 0.1 mg/mL streptomycin) for 30 min. Viral suspension was aspirated, and cells were washed with PBS and further incubated in infection media (DMEM_INF_: DMEM supplemented with 0.2% bovine serum albumin (BSA), 1 mM MgCl_2_, 0.9 mM CaCl_2_) up to 8 hpvi, 24 hpvi, 32 hpvi, 44 hpvi, or 48 hpvi (hours post-viral infection).

### 2.3. Transfection Protocol

For transfection of the 3× NFκB reporter plasmid construct as described elsewhere [22] (0.1 µg/µL) A549 cells were seeded in 12-well plates as described above. Cells were transfected with 0.1 µg/µL of the indicated plasmid for 4 h with Lipofectamine^®^ 2000 (Invitrogen, Carlsbad, CA, USA) corresponding to the manufacturer’s protocol. Afterwards, cells were washed with PBS and further incubated in cell culture media up to 24 h. Afterwards transfected cells were infected up to 8 hpvi. Performance of luciferase assay was done as described elsewhere [23].

### 2.4. Intra- and Extracellular Bacterial Titer Measurements

Extracellular bacterial titers were determined by collecting the supernatant of infected cells including the washing with PBS. Cells were lysed via hypotonic shock with 2 mL ddH_2_O according to Tuchscherr et al. [7,8] (37 °C, 30 min) to determine intracellular bacterial titers, including adherent bacteria at the cells surface. Bacterial suspensions were centrifuged (4000 rpm, 4 °C, 10 min), pellets were resuspended in 1 mL PBS, and serial dilutions (1:10) were plated on BHI agar plates and incubated for 32 h at 37 °C.

### 2.5. Standard Plaque Assay

Infectious virus particles in the supernatant were titrated to determine viral titers. A standard plaque assay was performed as described earlier [24].

### 2.6. Quantitative Real-Time PCR (qRT-PCR)

RNA isolation was performed with RNeasy Kit (Qiagen, Hilden, Germany) according to the manufacturer’s instructions. Reverse transcription was performed with 2 µg of total RNA with Revert AID H Minus Reverse Transciptase (Thermo Fisher Scientific, Karlsruhe, Germany) and oligo (dT) primers according to the manufacturer’s protocol. qRT-PCR was performed using a Roche LightCycler 480 and Brilliant SYBRGreen Mastermix (Agilent, Santa Clara, CA, USA) according to the manufacturer’s instructions. The following primers were used: GAPDH: fwd 5′GCAAATTCCATGGCACCGT3′, rev 5′GCCCCACTTGATTTGGAGG3′; IL-6: fwd 5′AACCTGAACCTTCCAAAGATGG3′, rev 5′TCTGGCTTGTTCCTCACTAGT3′; IL-8: fwd 5′CTTGTTCCACTGTGCCTTGGTT3′, rev 5′GCTTCCACATGTCCTCACAACAT3′; TNFα: fwd 5′-ATGAGCACTGAAAGCATGATC-3′, rev 5′-GAGGGCTGATTAGAGAGAGGT-3′; IL-1β: fwd 5′-CAGCTACGAATCTCCGACCAC-3′, rev 5′-GGCAGGGAACCAGCATCTTC-3′; IFNγ: fwd 5′AAACGAGATGACTTCGAAAAGCTG3′, rev 5′TGTTTAGCTGCTGGCGACAG3′; RIG-I: fwd 5′CCTACCTACATCCTGAGCTACAT3′, rev 5′TCTAGGGCATCCAAAAAGCCA3′; IFNβ: fwd 5′TCTGGCACAACAGGTAGTAGGC3′, rev 5′GAGAAGCACAACAGGAGAGCAA3′; MxA: fwd 5′GTTTCCGAAGTGGACATCGCA3′, rev 5′GAAGGGCAACTCCTGACAGT3′; OAS1: fwd 5′GATCTCAGAAATACCCCAGCCA3′, rev 5′AGCTACCTCGGAAGCACCTT3′. Relative changes in expression levels (n-fold) were calculated according to the 2^−ΔΔC*t*^ method [25].

Bacterial RNA was isolated with the RNeasy Protect Bacteria Mini Kit (Qiagen, Hilden, Germany), and cDNA synthesis was performed using QuantiTect Reverse Transcription Kit (Qiagen, Hilden, Germany) according to the manufacturer’s instructions. qRT-PCR was performed using a Roche LightCycler 480 (Basel, Switzerland) and Brilliant SYBRGreen Mastermix (Agilent, Santa Clara, CA, USA) according to the manufacturer’s instructions. The primers to determine the gene expression of *gyrB*, *aroE*, *arg*, *hla*, *sarA*, and *sigB* were already described elsewhere [7].

### 2.7. RT^2^ Profiler Array Analysis

For pathway focused gene expression analysis, we used *RT^2^ Profiler PCR Arrays* (Qiagen, Hilden, Germany). RNA isolation, cDNA synthesis and procedure were performed according to the manufacturer’s protocol and instructions. Analysis of data was accomplished by using the GeneGlobe Data Analysis Center recommended by Qiagen [26].

### 2.8. FACS Analysis

Determination of secreted proteins in the supernatant was performed with BioLegend’s LEGENDplex™ (San Diego, CA, USA) according to the manufacturer’s protocol. The human anti-viral and pro-inflammatory chemokine panels were used. Results were analyzed by BioLegend’s cloud-based LEGENDplex™ Data Analysis Software. To analyze apoptotic or necrotic cells, infection was performed as described above until 44 hpvi. Cells were treated with tumor necrosis factor related apoptosis inducing ligand (TRAIL; Enzo Life Sciences, Farmingdale, NY, USA) (150 ng/mL) 4.5 h before harvested and used as a positive control for apoptosis. The supernatant was collected for this purpose, and cells were detached from the wells with trypsin-EDTA and recombined with the supernatant. Cell suspension was centrifuged at 1000× *g* at room temperature (RT) for 5 min, and cells were washed with PBS supplemented with 5% FCS. Afterwards, cells were stained with annexin V FITC (20 µL) (ImmunoTool, Friesoythe, Germany) and 1:2000 eBioscience™ Fixable Viability Dye eFluor™ 660 (Thermo Fisher Scientific, Karlsruhe, Germany) in 100 µL 1× annexin V staining buffer (10× annexin V staining buffer: 0.1 M HEPES, 1.4 M NaCl, and 25 mM CaCl_2_ (pH 7.5)) for 30 min at RT in the dark. Further 150 µL of staining buffer were added and the supernatant was removed after centrifugation. Cells were fixed with 500 µL PBS containing 4% formaldehyde and 1.25 mM CaCl_2_ for 20 min at RT in the dark. Cells were finally resuspended in 150 µL staining buffer and stored at 4 °C until measurement with the FACSCalibur flow cytometer (BD Biosciences, Heidelberg, Germany), followed by the analysis with FlowJo software (v.10; Flow Jo, Ashland, OR, USA). Three gates were set as the following: annexin V positive cells (early apoptotic cells) and live/dead marker positive cells (cells with a membrane rupture tending to necrosis).

### 2.9. Recording of Cytopathic Effect of Infected Cells

To record the CPE at different time points, cells were visualized with Canon (EOS 500D) by light microscopy (Axiovert 40C, ZEISS, Jena, Germany) with a 10× magnification.

### 2.10. SDS-PAGE and Western Blot Analysis

Protein expressions were determined by separating proteins in a polyacrylamide gel and subsequent transfer on nitrocellulose membranes by western blot analysis as described earlier [27]. The following antibodies were used: pMLKL [(S353) #91689 Cell Signaling, Frankfurt, Germany], PARP (#611039 BD, Heidelberg, Germany) and ERK1/2 (#4696 Cell Signaling, Frankfurt, Germany).

### 2.11. Lactate Dehydrogenase (LDH) Assay

The lactate dehydrogenase assay (CellBiolabs, San Diego, CA, USA) was used to measure the cell cytotoxicity and was used according to the manufacturer’s instructions. Cells were infected as described previously, and 90 µL of the supernatant was mixed with 10 µL of the LDH cytotoxicity reagent in a 96-well plate. This plate was incubated at 37 °C and 5% CO_2_ for 30 min, and the OD_450nm_ was measured on a Spectromax M2 Instrument (Molecular Devices, Munich, Germany). Triton X-100 used according to the manufacturer’s instructions served as a positive control.

### 2.12. Quantification and Statistical Analysis

All data represent the means + standard deviation (SD) of three independent experiments. Statistical significances were determined by unpaired *t*-test (Figure S4A), one-way ANOVA followed by Tukey’s, (Figures 4D,E, 5, 6A,B,D,E, Figure S1B,D,E, S2D,E, S3 and S4C–E) or two-way ANOVA followed by Sidak’s (Figure 2, Figures S1C and S4B) or followed by Tukey’s (Figure 4A–C,F–I and Figure S2A–C,F–I) multiple comparison test using GraphPad Prism software (v.7.03, GraphPad Prism, Inc., La Jolla, CA, USA).

## 3. Results

### 3.1. Primary S. aureus 3878_SCV_ Infection Provokes a Cytopathic Effect in Presence of IV

Cell death mechanisms induced by *S. aureus* or IV alone are very well investigated and described [28,29,30,31,32,33]. With respect to IV and *S. aureus* super-infection, we recently were able to show a *S. aureus*-mediated switch from IV-induced apoptosis to necrosis [27]. It is known that IV infection paves the path for secondary bacterial infection, resulting in enhanced pathogen-load [15,34], cytokine expression [35,36], and cell death [27]. Since *S. aureus* often persist in humans without any harm, we aimed to investigate the effects of colonizing *S. aureus* SCVs on secondary IV super-infection.

**Figure 1:**
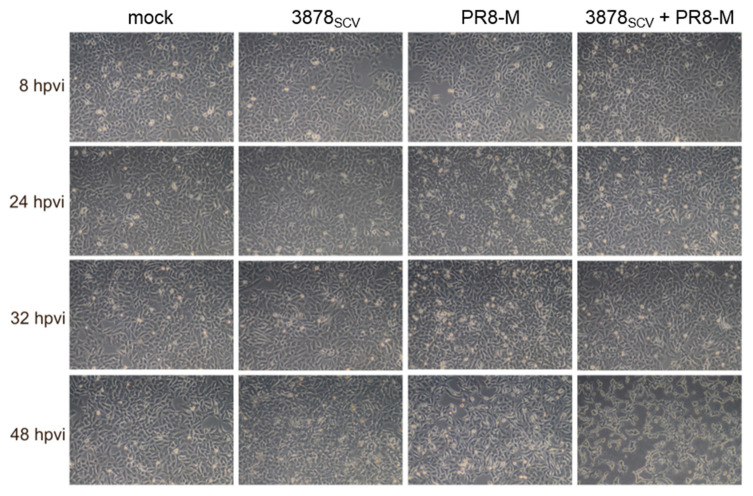
*S. aureus* 3878_SCV_ colonization and subsequent influenza virus infection provokes a cytopathic effect. A549 human lung epithelial cells were infected with *S. aureus* 3878_SCV_ (multiplicity of infection (MOI) = 0.01) for 24 h at 37 °C and 5% CO_2_. Afterwards, cells were infected with influenza viruses (IV) Puerto Rico/8 (PR8)-M (MOI = 0.1) until the indicated points in time (hours post-viral infection, hpvi). Cells were visualized by light microscopy with a 10× magnification. Shown are representative images of three independent experiments (*n* = 3).

In a first set of experiments, we focused on the cell morphology of A549 human lung epithelial cells in absence and presence of *S. aureus* 3878_SCV_ and IV. For this reason, A549 human lung epithelial cells were infected with *S. aureus* 3878_SCV_, which is a well described SCV patient isolate [10,37], for 24 h followed by infection with IV strain A/Puerto Rico/8/34 (PR8-M; H1N1) for the indicated points in time. The morphology of single- and super-infected cells was analyzed by light microscopy in comparison to uninfected control (mock) (Figure 1). While the cell monolayer is still intact in un-, single-, and super-infected cells up to 32 hpvi (hours post-viral infection), first changes in the cell morphology were visible 48 hpvi in single virus-infected and super-infected cells. Pictures of virus-infected cells showed a less confluent cell monolayer compared to uninfected cells, and in super-infected samples a clear cytopathic effect was observed, indicated by cell monolayer disruption and floating cells. To be able to ascribe these findings to the SCV phenotype, we additionally specified the pathological difference between *S. aureus* wildtype phenotype and SCV phenotype (*S. aureus* 3878_WT_ and *S. aureus* 3878_SCV_) by infecting A549 human lung epithelial cells. Cell morphology was monitored by light-microscopy (Supplementary Figure S1A) and cell viability was quantified by lactate dehydrogenase assay (LDH) assay (Supplementary Figure S1B). Both assays indicate a massive destruction of the cell monolayer 8 h post bacterial infection (hpbi) with *S. aureus* 3878_WT_ in comparison to *S. aureus* 3878_SCV_. Further, the determination of the expression of distinct bacterial genes, which are involved in the virulence of the pathogens, verified the reduced virulence of *S. aureus* 3878_SCV_ in comparison to the *S. aureus* 3878_WT_ (Supplementary Figure S1C). Based on these results, *S. aureus* 3878_WT_ was not used in the following experiments. The analysis of cell viability at 32 hpvi and 48 hpvi confirmed the cell disturbance in presence of *S. aureus* 3878_SCV_ and IV infection (Supplementary Figure S1D,E). 

These results point to an altered cell culture environment and/or cellular signaling upon super-infection with *S. aureus* 3878_SCV_ and IV, which could be triggered by increased pathogen load or cell intrinsic signaling changes in presence of both pathogens.

### 3.2. Primary Infection with S. aureus 3878_SCV_ Followed by IV Infection Had No Impact on Bacterial or Viral Titers

**Figure 2:**
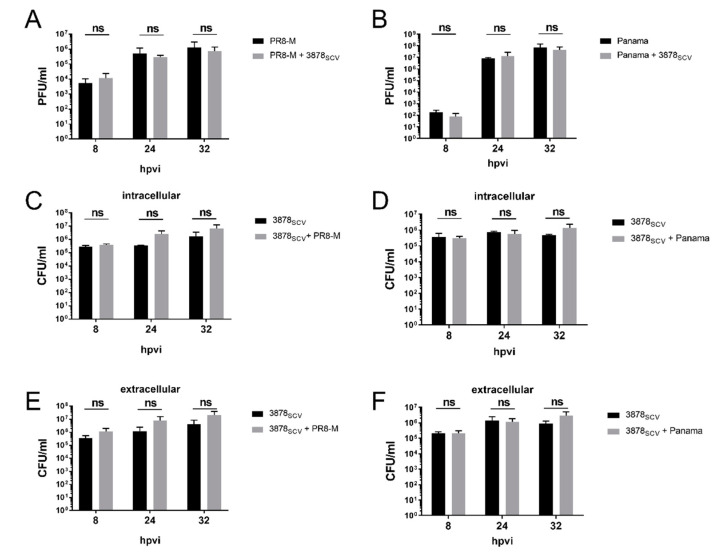
Pathogen load is not affected during *S. aureus* 3878_SCV_ colonization and subsequent influenza virus infection. A549 human lung epithelial cells were infected with *S. aureus* 3878_SCV_ (MOI = 0.01) for 24 h and/or super-infected with (**A**,**C**,**E**) IV PR8-M (H1N1; MOI = 0.1) or (**B**,**D**,**F**) IV Panama (H3N2; MOI = 0.01) for 8 hpvi, 24 hpvi, or 32 hpvi. At the indicated times post-viral infection, supernatants were collected to determine viral and extracellular bacterial titers. Afterwards, cells were lysed via hypotonic shock to analyze intracellular bacterial titers. Means + SD of three independent experiments with technical duplicates are shown (*n* = 3). Statistical significance (compared to single-IV infection (**A**,**B**) or single-bacteria infection (**C**–**F**) was analyzed by a two-way ANOVA, followed by Sidak’s multiple comparison test; (hpvi = hours post-viral infection; ns = not significant).

First, we analyzed whether the observed cytotoxicity of co-infected A549 human lung epithelial cells with *S. aureus* 3878_SCV_ and IV was due to increased pathogen load. For this, we infected A549 cells with *S. aureus* 3878_SCV_ for 24 h and super-infected with two different IV strains for the indicated points in time to determine the amount of plaque forming units (PFU) or colony forming units (CFU) of viruses or bacteria, respectively (Figure 2).

In general, titers of IV and SCVs increased with time, but neither viral (Figure 2A,B) nor bacterial titers (Figure 2C–F) were significantly changed upon super-infection compared to single-infected cells, a phenomenon independent of the virus strain used [PR8-M (H1N1), A/Panama/2007/99 (Panama; H3N2)]. 

Thus, these data indicate that the disruption of the cell monolayer upon super-infection is not induced by increased amounts of pathogens but by a different mechanism that is altered by the presence of both pathogens.

### 3.3. Pro-Inflammatory Gene Expression Is Highly Upregulated after Super-Infection of S. aureus 3878_SCV_ and IV

**Figure 3:**
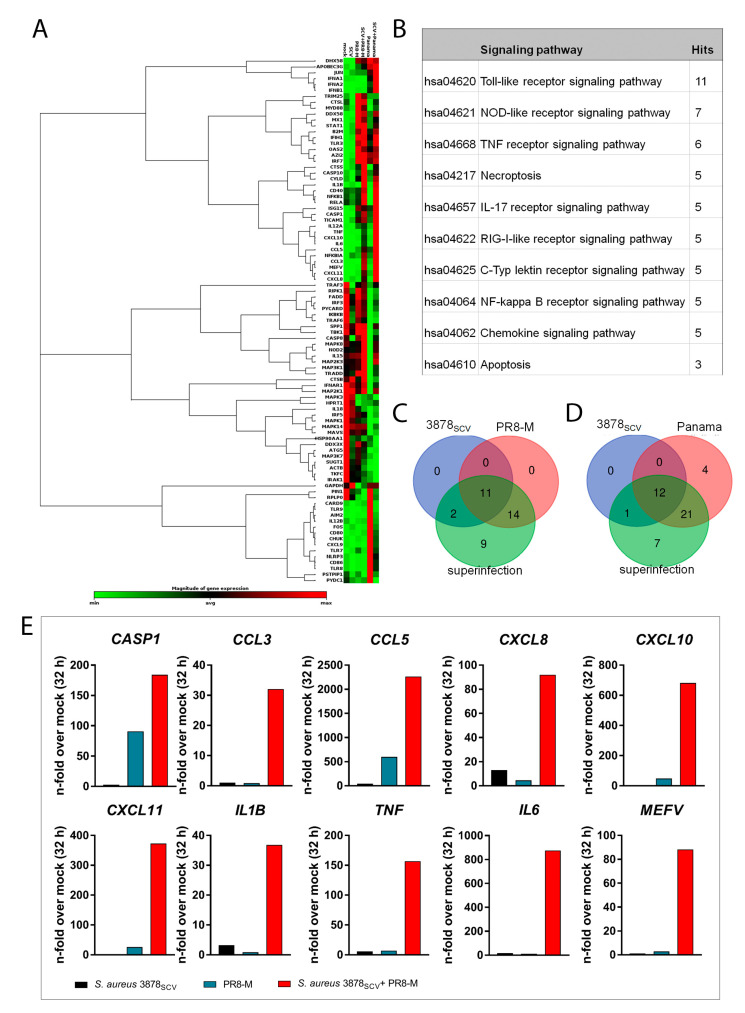
Gene expression analysis by *RT^2^ Profiler Array*. (**A**–**E**) A549 lung epithelial cells were infected with *S. aureus* 3878_SCV_ (MOI = 0.01) for 24 h and/or super-infected with IV PR8-M (H1N1; MOI = 0.1) or IV Panama (H3N2; MOI = 0.01) for 32 h. Subsequently, RNA was isolated and further used to perform the *RT*^2^ *Profiler Array* (Qiagen, Hilden, Germany). Ct-values were analyzed with the recommended QIAGEN web portal [26]. (**A**) A clustergram is shown, visualizing the up- and downregulated genes of the customized 84-gene array. (**B**) Ten potential signaling pathways are listed, which can be analyzed by the *RT^2^ Profiler Array* with the correlating count of genes involved. The mapping was done by using the Kyoto Encyclopedia of Genes and Genomes (KEGG) mapper [38,39,40]. (**C**,**D**) A Venn diagram of the upregulated genes in a super-infection scenario with *S. aureus* 3878_SCV_ and IV PR8-M (**C**) or IV Panama (**D**) is shown. The analysis was performed by use of http://bioinformatics.psb.ugent.be/webtools/Venn/. (**E**) Gene expression of highly induced genes indicating an increased pro-inflammatory cytokine response. Values are shown as n-fold over mock (32 hpvi); (*n* = 1); (hpvi = hours post-viral infection).

Given the observation that super-infection of *S. aureus* 3878_SCV_ and IV PR8-M induced a cytopathic effect (Figure 1), which was not caused by increased pathogen load (Figure 2), we aimed to elucidate if changes of cell intrinsic signaling and inflammatory gene expression might be responsible for this phenomenon. We analyzed the gene expression of 84 different genes, involved in different signaling cascades by use of a *RT^2^ profiler Array* (Qiagen, Hilden, Germany) in a single experiment to gain a first insight in the complexity of cellular signaling (Figure 3). This enables a quick analysis of expression levels of different genes that are organized by their function to be able to limit the amount of genes, altering the cell intrinsic signaling. Here, we used the anti-viral immune response panel, including pattern recognition receptors (PRRs), cytokines, and chemokines involved in pathogen recognition and immune responses. The bioinformatic analysis is based on conventional ct-values and was performed with the recommended GeneGlobe online software [26]. A clustergram was generated to visually illustrate all up- and downregulated genes that were analyzed (Figure 3A). To further interpret the results of the *RT*^2^ *profiler Array*, we did an in silico clustering of the upregulated genes of the array that were highly upregulated (difference of an n-fold of 2) in *S. aureus* 3878_SCV_ and PR8-M super-infected cells compared to single-infected cells (*APOBEC3G*, *CASP1*, *CASP10*, *CCL3*, *CCL5*, *CD40*, *CD80*, *CTSS*, *CXCL10*, *CXCL11*, *CYLD*, *IL1B*, *IL6*, *CXCL8*, *MEFV*, *TLR3*, *TNF*, *B2M*) (see Supplementary Table S1), with respect to their linkage to specific signaling pathways (Figure 3B) by using the Kyoto Encyclopedia of Genes and Genomes mapper (KEGG mapper). KEGG mapper is a database resource of collected information about pathways and the involved genes representing a pool of molecular interactions, reactions, and their relation to each other [38,39,40]. Down-regulated genes were excluded, as the gene expressions were negligible (Supplementary Table S1). Besides gene clusters connected to expected PRR pathways including TLR-(11 genes involved, out of the 18 highly upregulated genes comparing co- and single-infected cells identified (11/18), NLR- (7/18), TNFR- (6/18), RLR- (5/18), and NFκB- (5/18) signaling pathways (Figure 3B), we identified gene clusters belonging to two cell death mechanisms, necroptosis (5/18) and apoptosis (3/18). Furthermore, we identified genes involved in the IL-17 (5/18) and c-type lectin (5/18) signaling pathways. To further classify the activated genes leading to the observed cytopathic effect on human lung epithelial cells, we searched for a specific induction pattern in which super-infected cells led to upregulated genes. We, therefore, compared all upregulated genes of single-infected to super-infected samples in a Venn diagram (Figure 3C,D). We identified 11 genes that were induced in all three infection-scenarios compared to uninfected cells and 9 genes that were upregulated in super-infected cells only. We also compared the upregulated genes for super-infection with IV Panama. Here, all infection scenarios shared the induction of 12 genes, where 7 genes were exclusively induced by the super-infection of *S. aureus* 3878_SCV_ and IV Panama. The upregulated genes of the Venn diagram are listed in Table S2A,B. With respect to the mRNA expression levels shown in Supplementary Table S1 and the cytopathic effect observed in super-infected cells (Figure 1), an induction of pro-inflammatory immune response can be concluded, which was further visualized by graphs, exhibiting the gene expression of the highly upregulated genes (Figure 3E).

**Figure 4:**
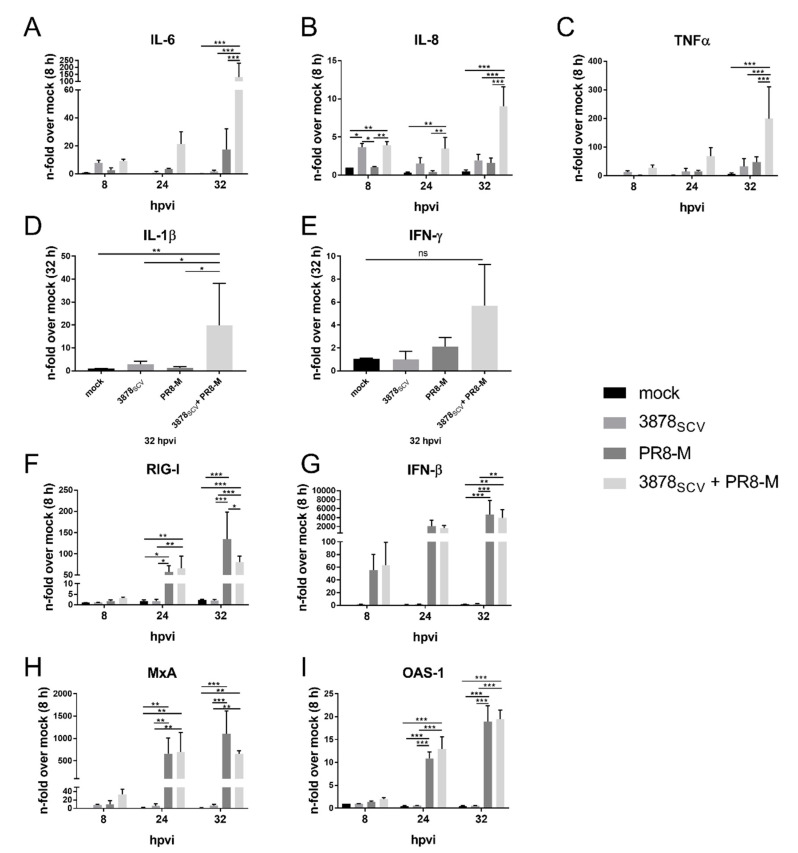
Pro-inflammatory cytokines and chemokines are enhanced after *S. aureus* 3878_SCV_ colonization and subsequent IV PR8-M infection. (**A**–**I**) A549 human lung epithelial cells were infected with *S. aureus* 3878_SCV_ (MOI = 0.01) for 24 h and/or super-infected with IV PR8-M (H1N1; MOI = 0.1) for 8 hpvi, 24 hpvi and/or 32 hpvi. Afterwards, RNA was isolated and mRNA levels of IL-6, IL-8, TNFα, IL-1β, IFN-γ, RIG-I, IFN-β, MxA, and OAS1 were determined by qRT-PCR. All values were correlated to the representative mock-control 8 hpvi (IL-6, IL-8, TNFα, RIG-I, IFNβ, MxA and OAS1) or 32 hpvi (IL-1β and IFN-γ). Means + SD of three independent experiments including technical duplicates are shown. Statistical significance was analyzed by a two-way (**A**–**C**), (**F**–**I**) or one-way (**D**,**E**) ANOVA, followed by Tukey’s multiple comparison test (* *p* < 0.05, ** *p* < 0.01, *** *p* < 0.001); (hpvi = hours post-viral infection; ns = not significant).

To confirm an increased pro-inflammatory status of the human lung epithelial cells upon super-infection, we analyzed the mRNA expression of different representative pro-inflammatory cytokines and chemokines (IL-6, IL-8, TNFα, IL-1β, and IFN-γ) in detail (Figure 4A–E). Furthermore, we analyzed the mRNA expression of molecules that are involved in the induction of the type-I-IFN signaling (RIG-I, IFN-β, MxA, and OAS1) (Figure 4F–I), since it was described that IV-induced type-I-IFN signaling had an impact on bacterial infections [41].

In *S. aureus* 3878_SCV_ colonized cells subsequently infected with IV PR8-M the mRNA expression of IL-6, IL-8, TNFα, and IL-1β 32 hpvi was induced if compared to uninfected cells or single-infected cells (Figure 4A–D) and IFN-γ showed the same tendency (Figure 4E). Single-infection of *S. aureus* 3878_SCV_ or IV PR8-M resulted in no significant induction of the mRNA expression 8 hpvi, 24 hpvi, or 32 hpvi, except for IL-8, which was significantly induced 8 hpvi in bacteria single-infected cells (Figure 4B). Nevertheless, this induction was abolished over time. Genes, encoding key proteins involved in the recognition, and induction of type-I-IFN signaling were upregulated in IV PR8-M infected cells 8 hpvi (IFN-β by tendency) (Figure 4G) or 24 hpvi (RIG-I, MxA and OAS1) (Figure 4F,H,I). Previous colonization with *S. aureus* 3878_SCV_ had no impact on IV-induced mRNA expression of factors linked to the type-I-IFN response, except for RIG-I at 32 hpvi, which was significantly decreased in super-infected cells. However, the enhanced RIG-I mRNA synthesis did not result in alterations of viral titers. Similar results were obtained upon super-infection with *S. aureus* 3878_SCV_ and IV Panama, indicating a virus-independent effect (Supplementary Figure S2A–I).

**Figure 5:**
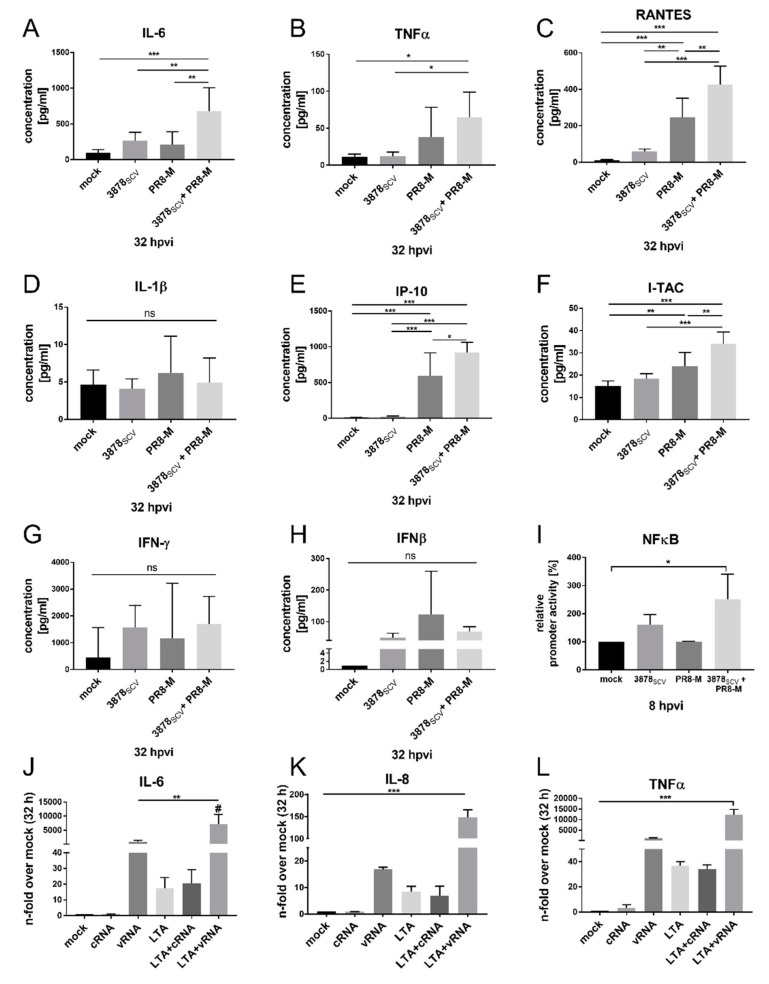
Secretion of the pro-inflammatory cytokines and chemokines are enhanced after *S. aureus* 3878_SCV_ colonization and subsequent IV PR8-M infection regulated by TLR2- and RIG-I-mediated NFκB promoter activation. (**A**–**H**) A549 human lung epithelial cells were infected with *S. aureus* 3878_SCV_ (MOI = 0.01) for 24 h and/or super-infected with IV PR8-M (H1N1; MOI = 0.1) for 32 h. Afterwards, supernatants were collected to measure the concentration of secreted proteins via FACS analysis. Means + SD of three independent experiments, including technical duplicates, are shown. (**I**) A549 human lung epithelial cells were transfected with 3× NFκB luciferase promoter reporter construct for 24 h prior to super-infection as described before. Afterwards cells were harvested and analyzed for luciferase activity. (**J**)–(**L**) A549 human lung epithelial cells were stimulated with LTA (100 ng/mL) for 24 h at 37 °C and 5% CO_2_. Afterwards, cells were stimulated with cellular RNA (cRNA) or viral RNA (vRNA) (100 ng/mL) in the presence or absence of LTA for 4 h at 37 °C and 5% CO_2_. Subsequently, RNA was isolated, and mRNA levels of IL-6, IL-8, and TNFα, were measured by qRT-PCR. All values are correlated to the respective mock-control (*n* = 3). Statistical significance was analyzed by one-way ANOVA followed by Tukey’s multiple comparison test (* *p* < 0.05, ** *p* < 0.01, *** *p* < 0.001; # = *** *p* < 0.001 compared to LTA + vRNA, except for vRNA); (hpvi = hours post-viral infection; ns = not significant).

To analyze whether the induction of mRNA synthesis of pro-inflammatory genes could also be detected on protein level and to get further insights into the cell intrinsic innate immune status of super-infected A549 cells, the protein expression of exemplary cytokines and chemokines was monitored by FACS analysis (Figure 5A–H). Remarkably, FACS analysis verified the increased pro-inflammatory response of A549 human lung epithelial cells for the secretion of representative factors. In super-infected cells, protein levels of IL-6, RANTES (CCL5), IP-10, and I-TAC were significantly induced compared to uninfected or single-infected cells with either *S. aureus* 3878_SCV_ or IV PR8-M (Figure 5A,C,E,F). TNFα was also significantly upregulated upon super-infection compared to uninfected and bacteria single-infected cells but not to IV PR8-M single-infected cells. Furthermore, IV PR8-M infection provoked TNFα protein expression 32 hpvi (Figure 5B). Representative IFN protein concentrations of IFN-γ and IFNβ (Figure 5G,H) showed no alteration in the amount of secreted proteins.

These results emphasize the enhanced cell intrinsic pro-inflammatory status of the super-infected cells. We could confirm these results by the use of IV strain Panama, verifying a viral strain independent effect (Supplementary Figure S3A–H). Based on these results and due to the fact that the induction of pro-inflammatory cytokines and chemokines is mainly driven by NFκB activation, we hypothesized an induction of the pro-inflammatory response via specific PRRs, resulting in the activation of the NFκB-signaling cascade [42]. To confirm the induction of NFκB-signaling we transfected A549 cells with an artificial NFκB promoter-dependent luciferase reporter plasmid prior to super-infection with *S. aureus* 3878_SCV_ and subsequent IV PR8-M infection. An increase of NFκB activation was observed in super-infected cells compared to uninfected and IV PR8-M-infected cells, while *S. aureus* 3878_SCV_ infection only resulted in an increase of NFκB activation by trend (Figure 5I). This induction pattern was also confirmed in cells super-infected with IV Panama (Supplementary Figure S3I).

The induction of pro-inflammatory responses via NFκB in epithelial cells after pathogen exposure can be triggered by both pathogens through different factors and their corresponding receptors [43,44,45], which were further analyzed. To exclude specific pathogen-mediated interference with cellular factors due to differences in protein expression, such as virulence factors or surface proteins, viral RNA (vRNA) and bacterial lipoteichonic acid (LTA, Invivogen, San Diego, CA, USA) were used as pathogen specific molecular stimuli. In human lung epithelial cells vRNA is mainly recognized by RIG-I, leading to a strong induction of the type-I-IFN-signaling cascade [46], while LTA is mainly recognized by TLR-2 [47]. We investigated mRNA expression of IL-6, IL-8, and TNFα after stimulating A549 cells with vRNA and LTA (Figure 5J–L). The results matched our findings obtained from super-infected cells, since significant enhancement of mRNA expression of IL-6, IL-8, and TNFα was observed in presence of both stimuli. Artificial effects caused by RNA transfection could be excluded due to equal cytokine mRNA expression induced by cellular RNA (cRNA) and cRNA + LTA stimulated cells. Stimulation with vRNA tended to induce the expression of IL-6, IL-8, and TNFα, which, however, was not significant compared to unstimulated cells.

Overall, these data suggest an induction of pro-inflammatory gene expression responses through the detection of bacterial and viral components via the pathogen-associated molecular pattern receptors (PAMP) RIG-I and TLR-2, followed by the induction of NFκB. To exclude bacterial strain-specific effects, another SCV strain (*S. aureus* 814_SCV_) was used to determine pathogen loads and pro-inflammatory gene expression in IV super-infection (Supplementary Figure S4). While neither viral titers nor intra- and extracellular bacterial load were increased in presence of both pathogens, pro-inflammatory cytokine expression was enhanced, verifying the former observations.

### 3.4. S. aureus 3878_SCV_ Provoke Enhanced Necrotic Cell Death in Presence of IV Infection

The observed disruption of the cell monolayer (Figure 1) could be induced by a variety of mechanisms. Besides the involvement of pro-inflammatory cytokines in the innate immune response, these factors are also involved in the induction of cell death mechanisms, like apoptosis and necrosis. As the results shown in Figure 3 indicate, an upregulation of pro-inflammatory cytokines, the cell death mechanisms might be triggered by TLRs or cell death receptors through PAMPs or cytokines, like TNFα, among others [48,49,50]. Therefore, we further investigated the induction of apoptosis and necrosis, correlating to the cell death mechanisms identified in the *RT^2^ Profiler Array* analysis (Figure 3B).

**Figure 6:**
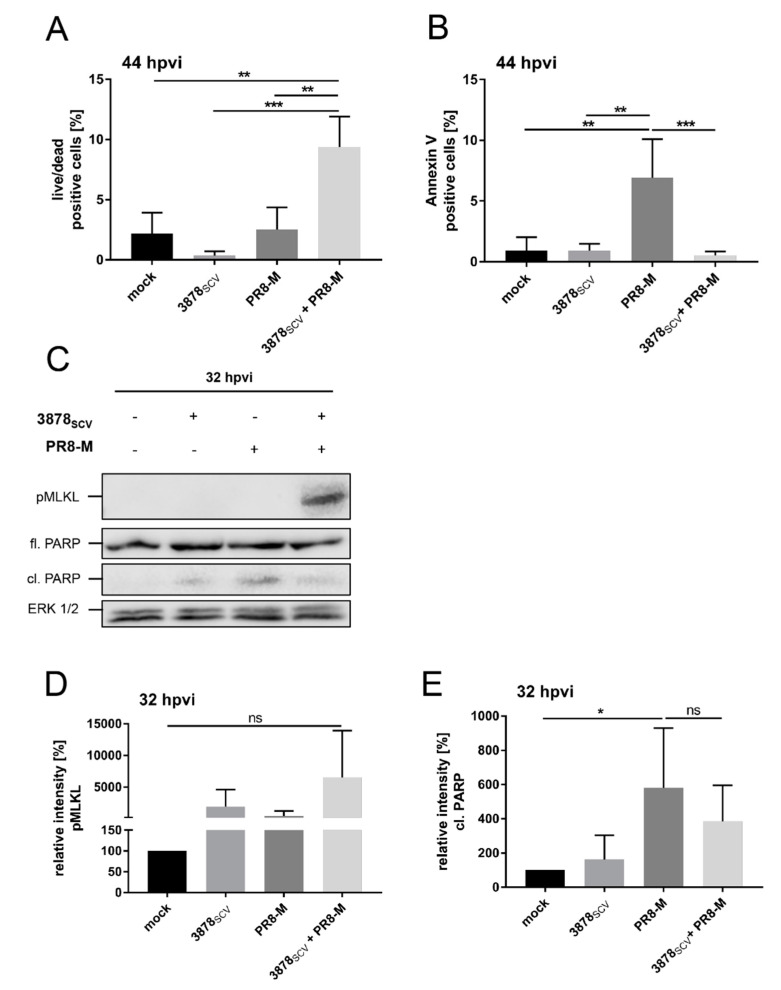
*S. aureus* 3878_SCV_ colonization and subsequent IV infection inhibits IV-induced apoptosis but results in the induction of necrosis. (**A**–**E**) A549 human lung epithelial cells were infected with *S. aureus* 3878_SCV_ (MOI = 0.01) for 24 h and/or super-infected with IV PR8-M (H1N1; MOI = 0.1) for 44 hpvi (**A**,**B**) or 32 hpvi (**C**–**E**). At the indicated times post-viral infection, total amount of cells was collected to perform FACS analysis to determine the relative amount of viability marker positive cells (**A**) or annexin V positive cells (**B**). Furthermore, whole cell lysates were subjected to western blot analysis (**C**). (**D**,**E**) Densitometrical analysis of three independent western blot experiments of cleaved pMLKL (**D**) and PARP (**E**) 32 hpvi are shown. Equal protein amounts were calculated by correlating the signal intensities to their corresponding ERK1/2 signals. Means + SD of three independent experiments are shown (*n* = 3). Statistical significance was analyzed by a one-way ANOVA, followed by Tukey’s multiple comparison test (**A**,**B**,**D**,**E**); (* *p* < 0.05, ** *p* < 0.01, *** *p* < 0.001); (hpvi = hours post-viral infection; ns = not significant).

As the results of the LDH assay led to the hypothesis of an induced necrotic cell death mechanism, we performed FACS analysis to determine the number of early apoptotic cells by detecting phosphatidylserine which switches to the cells’ surface in early apoptotic cells and can be labeled with annexin V. Cells with a membrane rupture tending to necrosis were detected by using a viability marker comparable to 7-aminoactinomycin D and propidium iodide staining. Therefore, we performed the infection up to 44 hpvi to be able to still distinguish between early apoptosis and necrotic-like cells and stained the cells accordingly. The amount of necrotic cells significantly increased comparing un- or single-infected with super-infected cells, probably indicating necrosis (Figure 6A). Furthermore, the amount of apoptotic cells was significantly higher in IV-infected cells compared to un-, bacteria-, or super-infected cells 44 hpvi (Figure 6B). 

As cell death mechanisms like necrosis can be further defined in specific mechanisms and to compare our findings to previously described inductions of cell death mechanisms upon infection with SCV [51] or in co-infection scenarios [27], we performed western blot analysis to be able to differentiate between necroptosis and apoptosis. 

Necroptosis is an inflammatory programmed form of necrosis, which was already described in a recent publication reporting its induction by *S. aureus* SCV in single-infected human primary keratinocytes [51]. Necroptosis is induced via a receptor-interacting protein (RIP) kinase-mediated activation, resulting in the phosphorylation and oligomerization of mixed lineage kinase domain like pseudokinase (MLKL) and pore formation, leading to the release of inflammatory cytokines. To distinguish necroptosis from apoptosis induction, we monitored the induction of phosphorylated MLKL and PARP cleavage, which are indications for both cell death mechanisms [52]. We infected A549 human lung epithelial cells with *S. aureus* 3878_SCV_ for 24 h, followed by IV infection with PR8-M for 32 h (Figure 6C–E). 

In super-infected cells, induction of pMLKL 32 hpvi was observed in comparison to uninfected, bacteria-, or virus single-infected cells, respectively. PARP cleavage was more likely to be induced in IV PR8-M-infected cells, and was slightly decreased in super-infected samples 32 hpvi (Figure 6C). However, the densitometrical analysis of three independent experiments could only confirm a trend of activated MLKL due to induced phosphorylation in super-infected cells (Figure 6D), whereas an induction of apoptosis upon IV infection could be verified (Figure 6E). Additionally, pyroptosis as another form of regulated necrotic cell death mechanism, which is activated via the induction of the inflammasome resulting in the cleavage of gasdermin D, could not be detected by cleaved gasdermin D (Supplementary Figure S5). The original blots are shown in the Supplementary Figure S6.

Thus, our results indicate a necrotic cell death induction, most likely induced by increased pro-inflammatory gene expression response after super-infection with *S. aureus* 3878_SCV_, followed by secondary IV infection with PR8-M and Panama.

## 4. Discussion

The first occurrence of persisting bacteria or SCVs was already described about 100 years ago [53]. Even though they are known for such a long time, not many studies were undertaken to elucidate their impact on cellular responses or their impact on additional infections with other pathogens. Our aim was to investigate the interaction of *S. aureus* SCVs with a subsequent IV infection in respect to epithelial cell responses, which built the first cellular barrier for pathogens in the lung. Here, we demonstrate that invasive *S. aureus* SCVs do have an impact on the cell intrinsic response in human lung epithelial cells, as indicated by highly secreted pro-inflammatory cytokines and chemokines (Figure 5 and Supplementary Figure S3) and, furthermore, an induction of necrotic cell death of super-infected compared to single-infected cells (Figure 6). This was somehow surprising, since the majority of SCVs are not described to significantly induce cell intrinsic responses, due to decreased secretion of virulence factors [54], a feature that would match their dormant status. In particular, not much is known about the impact of SCVs on lung tissue responses and nothing so far about their impact on a secondary IV infection. In this study, we were able to show a cytopathic effect accompanied by increased pro-inflammatory cytokine and chemokine release and necrotic cell death through colonizing *S. aureus* 3878_SCV_ and different IV strains, such as PR8-M and Panama.

Typically, super-infections with pathogenic *S. aureus* strains and IV led to increased pathogen loads accompanied with the induction of pro-inflammatory responses [13,35]. However, this could not be confirmed within the present study in the SCV and IV super-infection scenario. It was shown previously that super-infection with pathogenic *S. aureus* leads to the enhancement of viral titers due to the inhibition of STAT1 and STAT2 dimerization, resulting in decreased production of anti-viral factors [15]. This inhibitory effect could be excluded since mRNA expression of RIG-I, IFNβ, MxA, or OAS1 in super-infected cells compared to IV-infected cells were not altered (Figure 4 and Supplementary Figure S2). Based on these results, we could exclude an effect of the anti-viral response and the involvement of an altered pathogen load. Nevertheless, we identified a clear induction of pro-inflammatory cytokines and chemokines in human lung epithelial cells. Besides the attraction of immune cells and the induction of an anti-pathogen status of the cell, pro-inflammatory cytokines induce a stress response leading to the induction of cell death mechanisms via TLRs or death receptors [55,56,57].

To proof the impact of two main cell death mechanisms we performed FACS analysis to monitor early apoptotic and necrotic-like cells. Correlating to the LDH assays, we could confirm an increase in necrosis during super-infection (Figure 6A). Besides, the amount of apoptotic cells was decreased in super- compared to IV-infected cells. Further specifications of cell-death mechanisms by western blot analysis revealed the tendency for an increase of phosphorylated MLKL in super-infected cells, giving the hit of probably induced necroptosis. Concomitantly, IV-induced PARP cleavage was reduced in super-infected cells compared to IV PR8-M-infected cells by trend.

Interestingly, there are two different mechanisms described, how pathogenic *S. aureus* and *S. aureus* SCVs are able to induce necroptosis [27,51]. During the critical phase of *S. aureus* infection the virulence factor *agr* is induced [8], resulting in possible secretion of different toxins, which induces necroptosis [27,33]. In SCV-infected keratinocytes, necroptosis was driven by the activation of glycolysis [51]. *S. aureus* adopts its whole metabolism to persist within the host. The metabolic changes of *S. aureus* were already described elsewhere [58]. As the utilization of the tricarboxylic acid cycle for the host cell and the persisting bacteria is decreased, the glycolysis is stronger induced to generate adenosine triphosphate (ATP). As we observed a disruption of cell monolayer and a possible induction of phosphorylated MLKL upon super-infection, we linked our findings more to necroptosis (Figure 1 and Figure 6). Even though *S. aureus*-induced necroptosis might be independent of TLR stimulation [59], our data indicate a synergistic effect of *S. aureus* 3878_SCV_ and IV inducing cell death, which can be related to TLR2- and RIG-I-mediated pro-inflammatory response induction. In addition, our data show that the superinfection could be imitated with the stimuli LTA and vRNA. This underlines that the initial induction of the pro-inflammatory response and the subsequent cell death must be different from that of pathogenic bacterial strains that induce cell death much more quickly. In case of SCV, this indicates a lower virulence probably due to the decreased secretion of virulence factors. Nonetheless, dormant SCVs can work synergistically and affect the virus-induced immune response. As we performed pure ligand experiments, inhibitory effects of molecules of this pro-inflammatory cell intrinsic response is supposed to trigger cellular stress in the form of reactive oxygen species [60]. 

So far, the impact of *S. aureus* SCVs with subsequent IV infection had not been investigated. Interestingly, we could give first insights in this super-infection scenario and unravel one extraordinary role of a SCV patients’ isolate *S. aureus* 3878_SCV_ with subsequent IV infection. We observed an induction of pro-inflammatory cytokines and chemokines, which underlines the severity of the coincident occurrence of *S. aureus* SCVs and IV. These data point to a cross-interaction of necrotic cell death and pro-inflammatory cell intrinsic response, as the pathogens alone can induce an inflammatory response through PAMPs and secreted cell damage-associated molecular patterns (DAMPs). Upon necrotic cell death induction, further pro-inflammatory responses are induced via DAMP receptors [50], leading to an enhancement of pro-inflammatory cytokines and chemokines seen on transcriptional and translational level.

In summary, we were able to show that persistent *S. aureus* SCV and subsequent IV infection affects cell-internal immune response by inducing the release of pro-inflammatory cytokines and chemokines, resulting in cell death induction.

## Supplementary Materials

The following are available online at https://www.mdpi.com/2076-2607/8/12/1998/s1, Table S1: List of ct-values analyzed by GeneGlobe online software of the *RT^2^ Profiler Array* plate. Table S2: (A,B) Listed gene names of the venn diagrams shown in Figure 3. Figure S1: Wildtype phenotype *S. aureus* 3878 is more virulent compared to *S. aureus* 3878_SCV_, but *S. aureus* 3878_SCV_ induces LDH release upon super-infection with PR8-M. Figure S2: Pro-inflammatory cytokines and chemokines are enhanced after *S. aureus* 3878_SCV_ colonization and subsequent IV Panama infection. Figure S3: Secretion of the pro-inflammatory cytokines and chemokines are enhanced after *S. aureus* 3878_SCV_ colonization and subsequent IV Panama infection regulated by TLR2- and RIG-I-mediated NFκB promoter activation. Figure S4: Pathogen load and pro-inflammatory cytokines and chemokines are enhanced after super-infection with the SCV strain *S. aureus* 814_SCV_. Figure S5: *S. aureus* 3878_SCV_ colonization and subsequent influenza virus infection has no effect on the induction of pyroptosis. Figure S6: Original western blots of Figure 6C and S5A.

## Abbreviations

DAMP	damage-associated molecular pattern
hpbi	hours post bacterial infection
hpvi	hours post-viral infection
I-TAC	interferon-inducible T-cell alpha chemoattractant
IFN	interferon
IL	interleukin
IP-10	interferon gamma-induced protein 10
IV	influenza virus
LDH	Lactate dehydrogenase
MAPK	mitogen-activated protein kinase
MxA	interferon-induced GTP-binding protein MxA
NFκB	nuclear factor kappa-light-chain-enhancer of activated B-cells
NLR	NOD-like receptor
NOD	nucleotide-binding oligomerization domain
OAS1	2′-5′-oligoadenylate synthetase 1
PAMP	pathogen associated molecular pattern
PRR	pattern recognition receptor
RANTES	CC-chemokine ligand 5
RELA	nuclear factor NF-kappa-B p65 subunit
RIG-I	retinoic acid inducible gene I
RLR	RIG-I like receptor
*S. aureus*	*Staphylococcus aureus*
SCVs	small colony variants
TLR	toll-like receptor
TNFR	tumor necrosis factor receptor
TNFα	tumor necrosis factor alpha
