# Supplementary material for: Impact of Staphylococcus aureus Small Colony Variants on Human Lung Epithelial Cells with Subsequent Influenza Virus Infection
Source: Microorganisms. 2020 Dec 15;8(12):1998. doi: 10.3390/microorganisms8121998 (PMC7765246; doi:10.3390/microorganisms8121998)
Supplement: Supplementary file 1 [file microorganisms-08-01998-s001.zip › microorganisms-1018812-s/Supplemental material.docx]

Supplementary information

Table S1: List of ct-values analyzed by GeneGlobe online software of the *RT^2^ Profiler Array* plate. Table shows the ct-values correlated to uninfected samples (n = 1).

Table S2: (A, B) Listed gene names of the venn diagrams shown in Figure 3. (A) Gene names correspond to Figure 3 C. (B) Gene names correspond to Figure 3D.

Figure S1: Wildtype phenotype *S. aureus* 3878 is more virulent compared to *S. aureus* 3878_SCV_, and provokes LDH release at early times upon super-infection with PR8-M. (A, B) A549 human lung epithelial cells were infected with *S. aureus* 3878_SCV_ (MOI = 0.01) or *S. aureus* 3878_WT_ (MOI = 0.01) for 8 h at 37 °C and 5 % CO_2_. Cells morphology was visualized by light microscopy with a 10x magnification. Shown are exemplary images of three independent experiments (A) and the release of relative LDH (B). (C) Total RNA of over-night cultures of *S. aureus* 3878_SCV_ or *S. aureus* 3878_WT_ were isolated to determine by qRT-PCR the gene induction of *agr*, *hla*, *sarA* and *sigB*. (D, E) A549 human lung epithelial cells were infected with *S. aureus* 3878_SCV_ (MOI = 0.01) for 24 h at 37°C and 5 % CO_2_. Afterwards, cells were infected with IV PR8-M (MOI = 0.1) until 32 hpvi or 48 hpvi (hours post viral infection = hpvi). Relative LDH release was measured via photometer at OD 450 nm. Means + SD of three independent experiments with technical duplicates are shown (B-E) (n = 3). One-way ANOVA followed by Tukey’s multiple comparison test (B, D, E) or two-way ANOVA followed by Sidak’s multiple comparison test (*S. aureus* 3878_WT_ as reference) were performed for statistical significance (C); (* p < 0.05, ** p < 0.01, *** p < 0.001); (hpvi = hours post viral infection; hpbi = hours post bacterial infection; ns = not significant).

Figure S2: Pro-inflammatory cytokines and chemokines are enhanced after *S. aureus* 3878_SCV_ colonization and subsequent IV Panama infection. (A-I) A549 human lung epithelial cells were infected with *S. aureus* 3878_SCV_ (MOI = 0.01) for 24 h and/or super-infected with IV Panama (H3N2; MOI = 0.01) for 8 hpvi, 24 hpvi and/or 32 hpvi. Afterwards mRNA levels of IL-6, IL-8, TNFα, IL-1β, IFN-γ, RIG-I, IFN-β, MxA and OAS1 were determined by qRT-PCR post viral infection. All values were correlated to the representative mock-control 8 hpvi (IL-6, IL-8, TNFα, RIG-I, IFN-β, MxA and OAS1) or 32 hpvi (IL-1β and IFN-γ). Means + SD of three independent experiments including technical duplicates are shown (n = 3). Two-way (A-C, F-I) or one-way ANOVA (D, E) followed by Tukey’s multiple comparison test were performed for statistical significance (* p < 0.05, ** p < 0.01, *** p < 0.001); (hpvi = hours post viral infection; ns = not significant).

Figure S3: Secretion of the pro-inflammatory cytokines and chemokines are enhanced after *S. aureus* 3878_SCV_ colonization and subsequent IV Panama infection regulated by TLR2- and RIG-I-mediated NFκB promoter activation. (A-H) A549 human lung epithelial cells were infected with *S. aureus* 3878_SCV_ (MOI = 0.01) for 24 h and/or super-infected with IV Panama (H3N2; MOI = 0.01) for 32 hpvi. Afterwards supernatants were collected to measure the concentration of secreted proteins via FACS analysis. Means + SD of three independent experiments with technical duplicates are shown (n = 3). Statistical significance was analysed by one-way ANOVA followed by Tukey’s multiple comparison test (* p < 0.05, *** p < 0.001); (hpvi = hours post viral infection; ns = not significant).

Figure S4: Pathogen load and pro-inflammatory cytokines and chemokines are enhanced after super-infection with the SCV strain *S. aureus* 814_SCV_. (A-E) A549 human lung epithelial cells were infected with *S. aureus* 3878_SCV_ (MOI = 0.01) for 24 h and/or super-infected with IV PR8-M (H1N1; MOI = 0.01) for 32 hpvi. Afterwards, supernatants were collected to determine viral (A) and extracellular bacterial titers (B). Cells were lysed via hypotonic shock to analyze intracellular bacterial titers (B). The mRNA levels of IL-6, IL-8 and TNFα were determined by qRT-PCR 32 hpvi. All values were correlated to the representative mock-control 32 hpvi. Means + SD of three independent experiments including technical duplicates are shown (n = 3). Statistical significance was analysed by unpaired t-test (A) or two-way ANOVA followed by Sidak’s (B – compared to single-bacteria infection) or one-way ANOVA followed by Tukey’s (C-E) multiple comparison test (* p < 0.05, ** p < 0.01, *** p < 0.001); (hpvi = hours post viral infection; ns = not significant).

Figure S5: *S. aureus* 3878_SCV_ colonization and subsequent influenza virus infection has no effect on the induction of pyroptosis. A549 human lung epithelial cells were infected with *S. aureus* 3878_SCV_ (MOI = 0.01) for 24 h and/or super-infected with IV PR8-M (H1N1; MOI = 0.1) for 32 hpvi. Whole cell lysates were subjected to perform western blot analysis. One representative blot out of three independent experiments is shown (#97558, Cell Signaling). ERK1/2 served as loading control.

Figure S6: Original western blots of Figure 6 C and S5 A. (A, B) Lane 1: mock, lane 2: *S. aureus* 3878_SCV_ infection, line 3: IV infection, line 4: super-infection. Figure S6 A (32 hpvi) represent the original blots of Figure 6 C and Figure S6 B represents the original blots of Figure S5 A.
