# Supplementary figures and images for: Impact of Staphylococcus aureus Small Colony Variants on Human Lung Epithelial Cells with Subsequent Influenza Virus Infection
Source: Microorganisms. 2020 Dec 15;8(12):1998. doi: 10.3390/microorganisms8121998 (PMC7765246; doi:10.3390/microorganisms8121998)

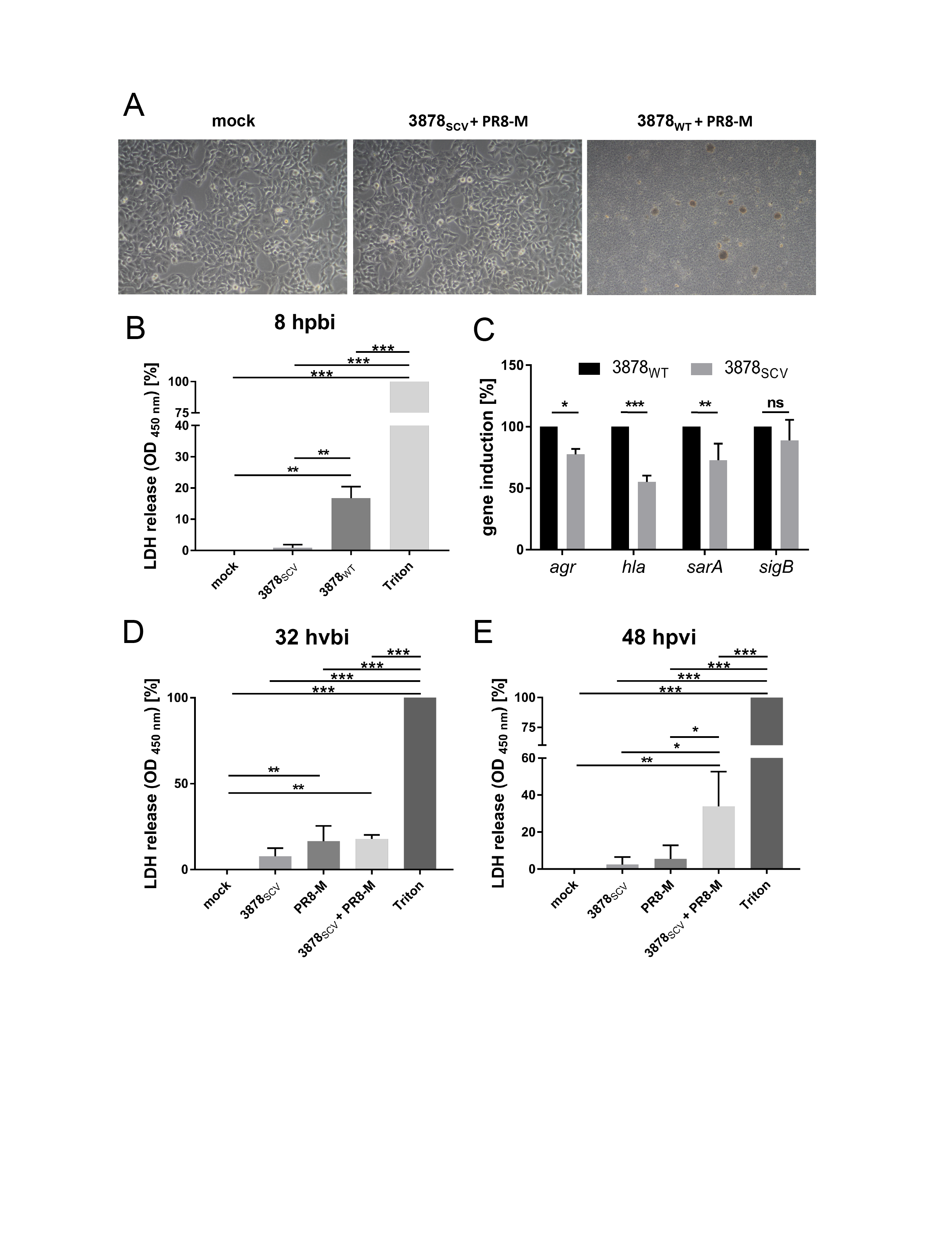

Supplement: Supplementary file 1 [file microorganisms-08-01998-s001.zip › microorganisms-1018812-s/Figure S1.tif]

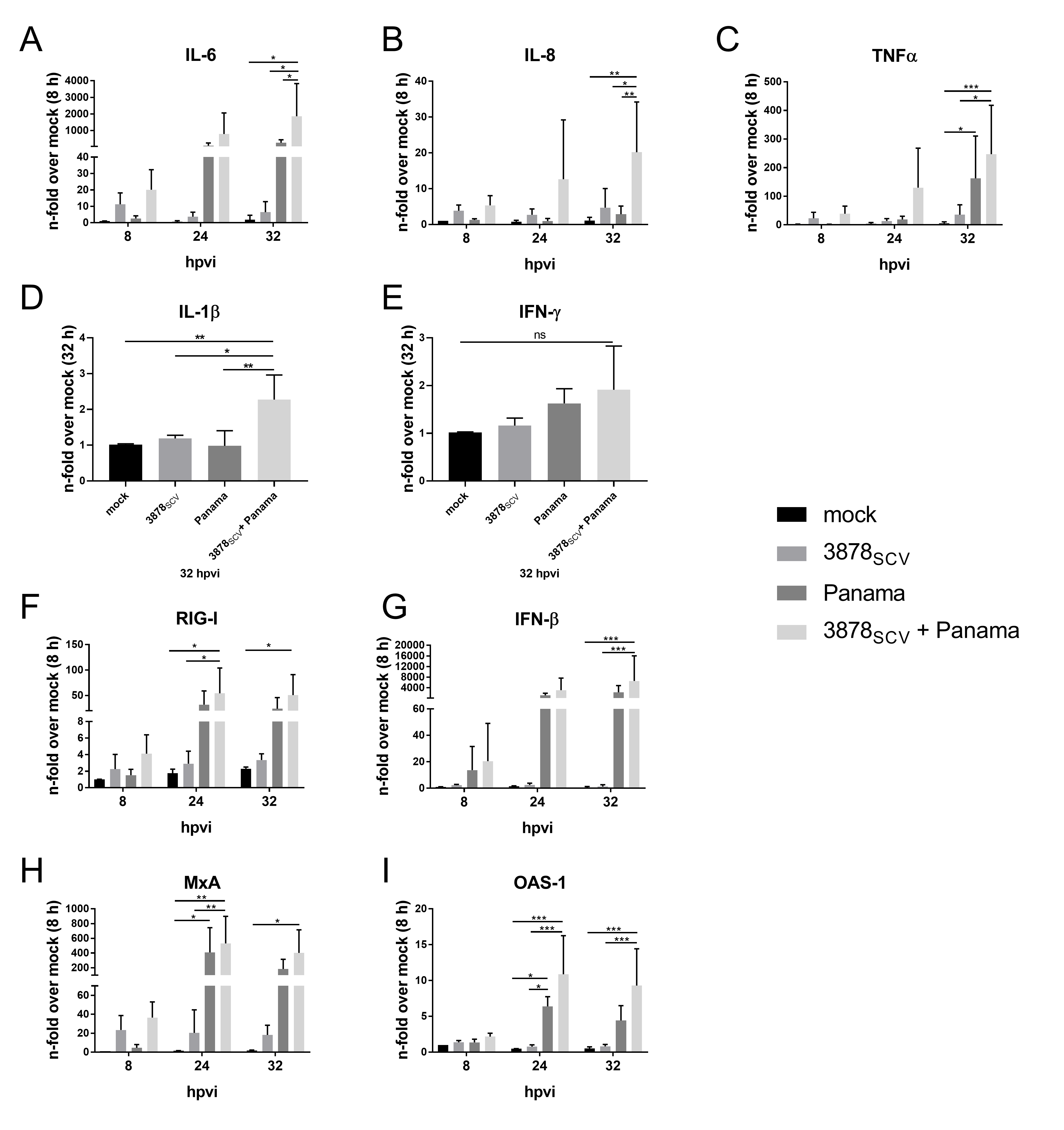

Supplement: Supplementary file 1 [file microorganisms-08-01998-s001.zip › microorganisms-1018812-s/Figure S2.tif]

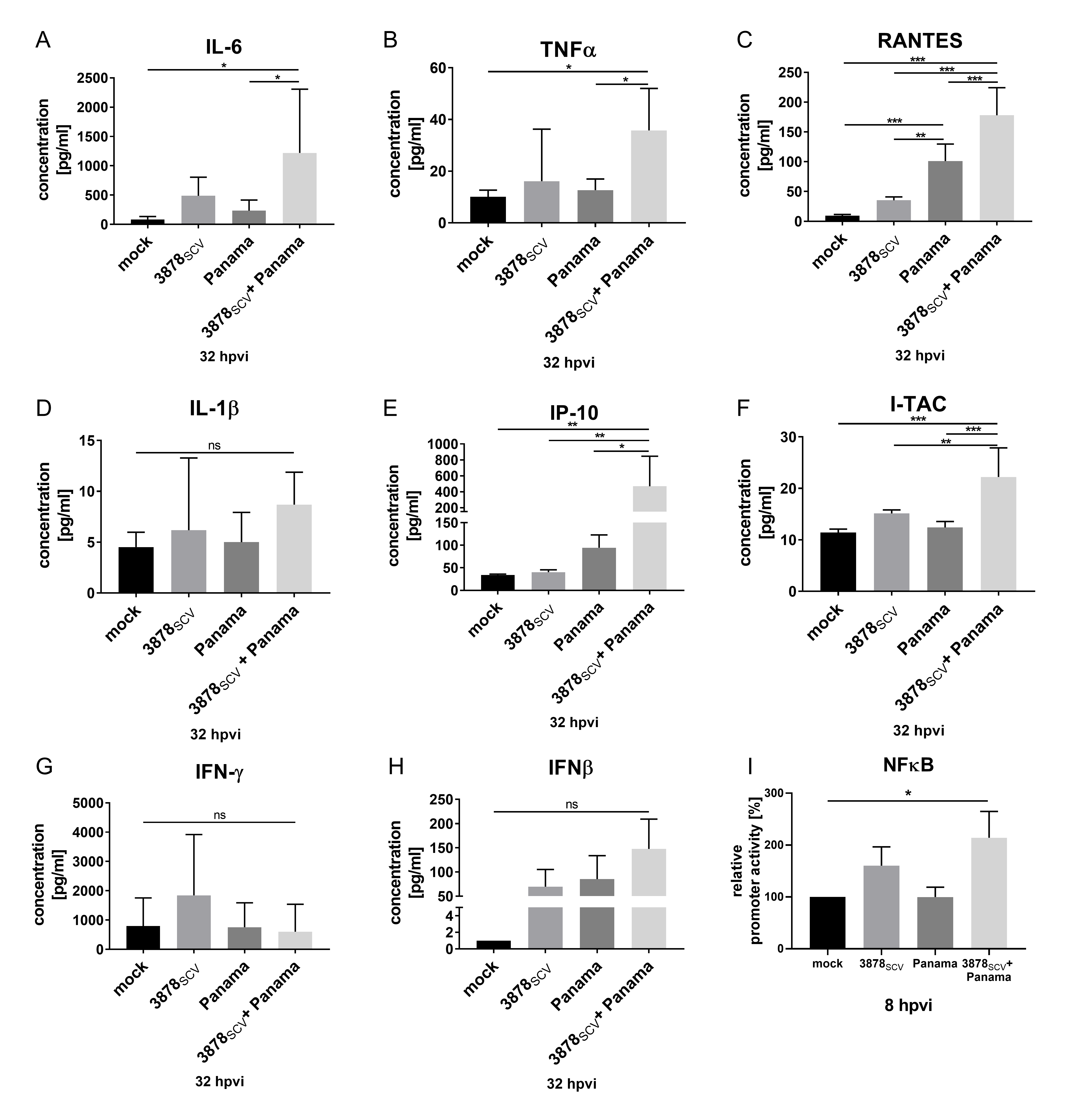

Supplement: Supplementary file 1 [file microorganisms-08-01998-s001.zip › microorganisms-1018812-s/Figure S3.tif]

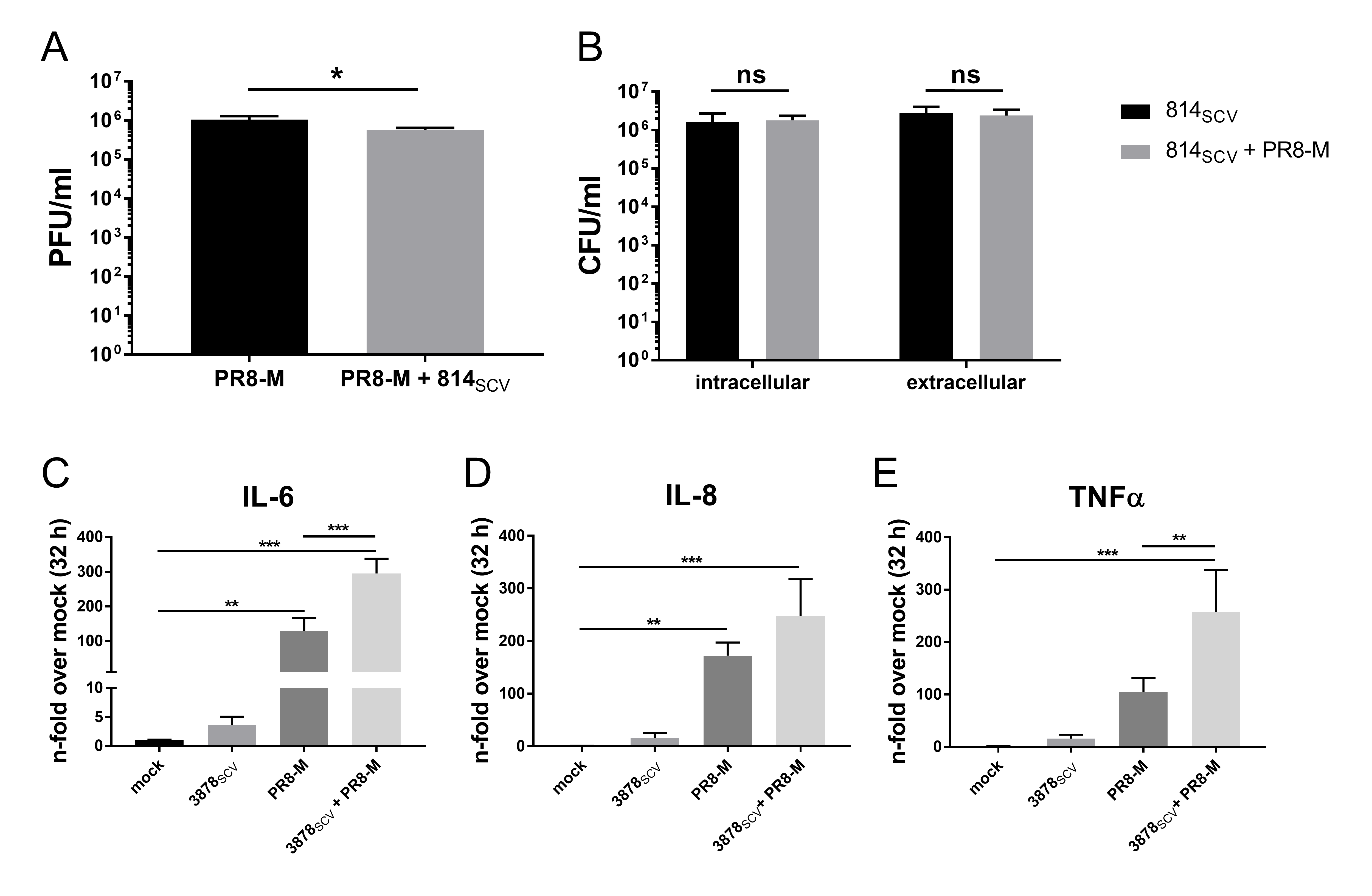

Supplement: Supplementary file 1 [file microorganisms-08-01998-s001.zip › microorganisms-1018812-s/Figure S4.tif]

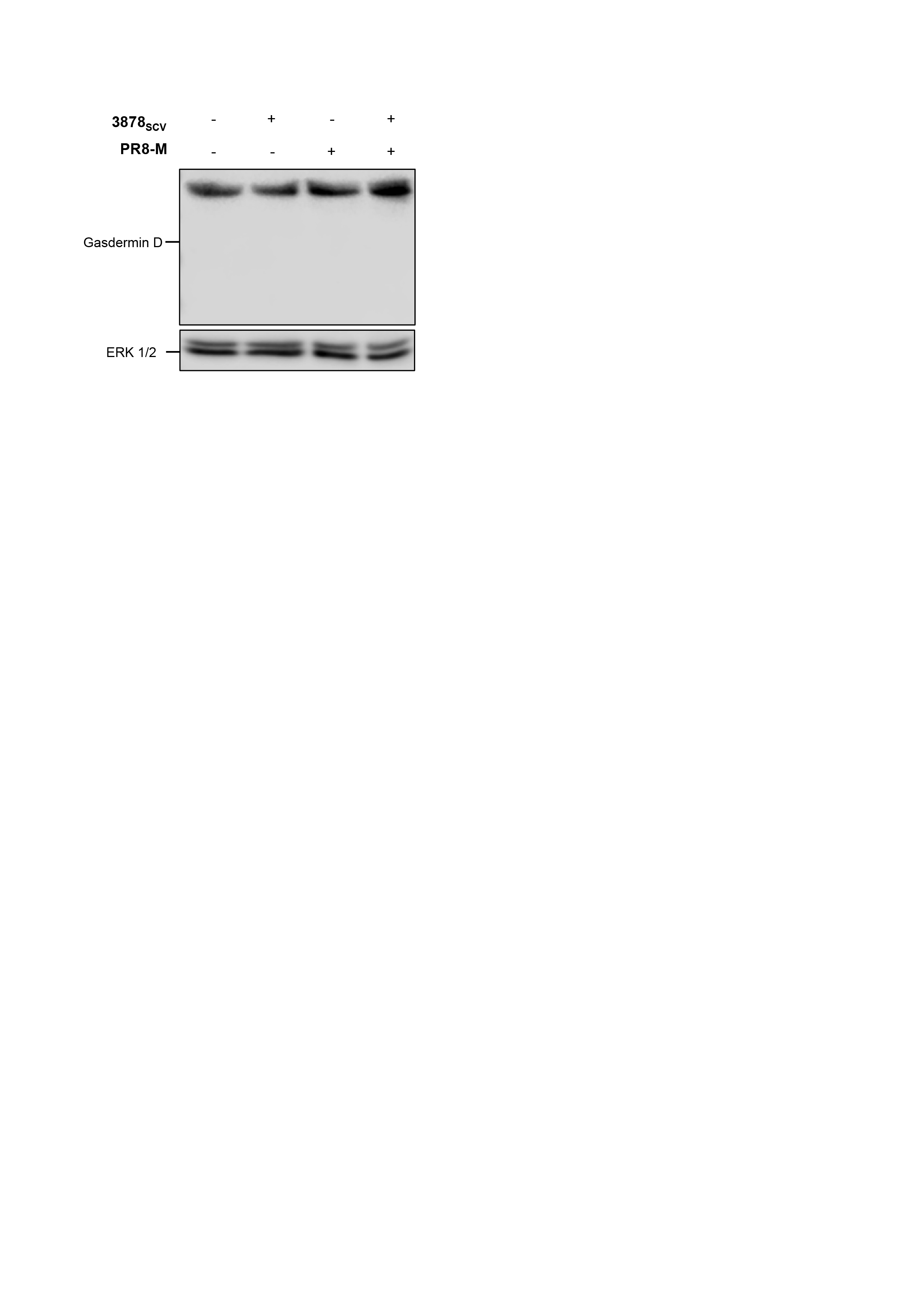

Supplement: Supplementary file 1 [file microorganisms-08-01998-s001.zip › microorganisms-1018812-s/Figure S5.tif]

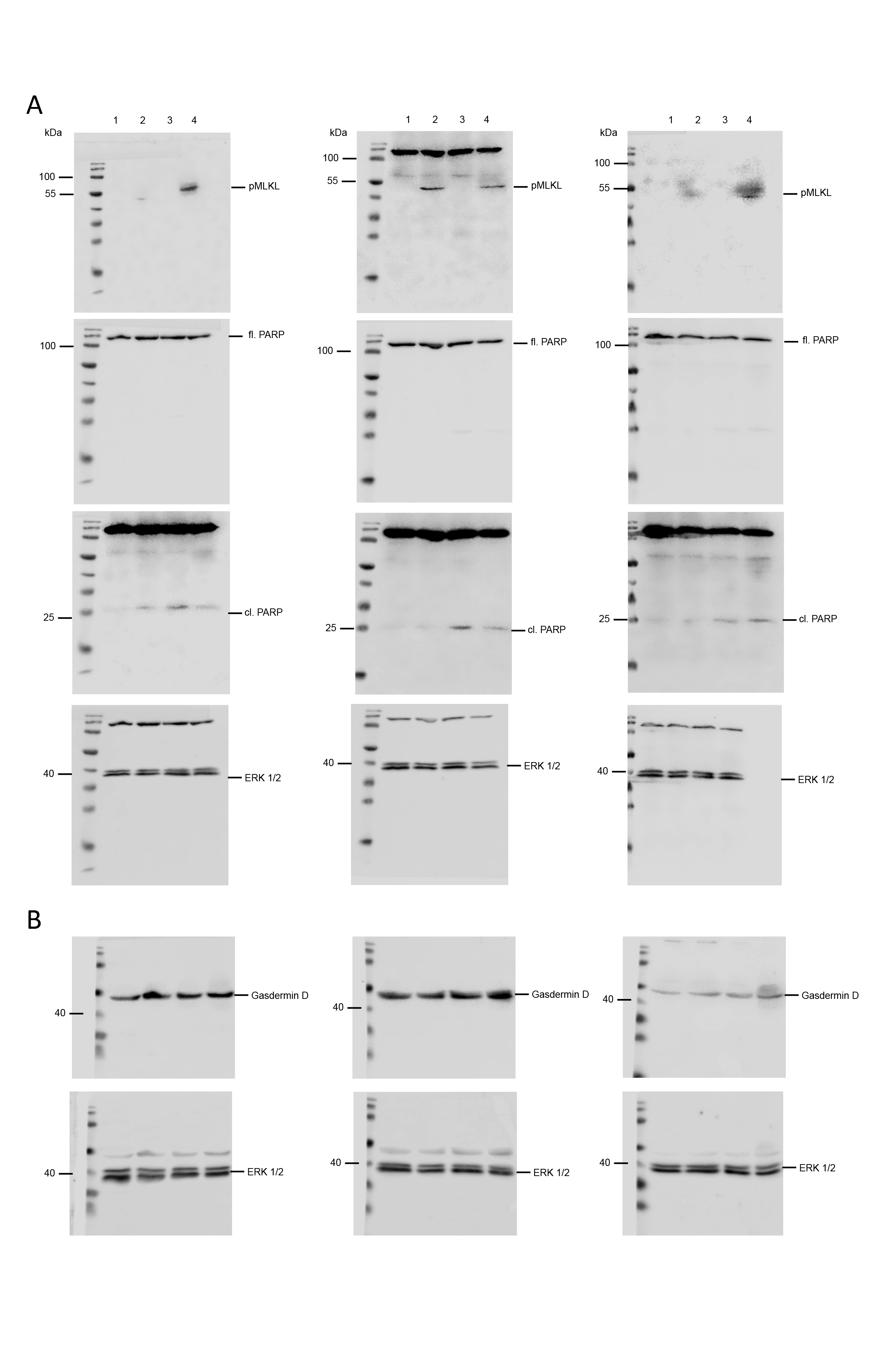

Supplement: Supplementary file 1 [file microorganisms-08-01998-s001.zip › microorganisms-1018812-s/Figure S6.tif]
